# Protein Engineering and High-Throughput Screening by Yeast Surface Display: Survey of Current Methods

**DOI:** 10.1002/smsc.202300095

**Published:** 2023-11-08

**Authors:** Joanan Lopez-Morales, Rosario Vanella, Elizabeth A. Appelt, Sarah Whillock, Alexandra M. Paulk, Eric V. Shusta, Benjamin J. Hackel, Chang C. Liu, Michael A. Nash

**Affiliations:** Institute for Physical Chemistry, Department of Chemistry, University of Basel, Basel 4058, Switzerland; Swiss Nanoscience Institute, University of Basel, Basel 4056, Switzerland; Department of Biosystems Science and Engineering, ETH Zurich, Basel 4058, Switzerland; Institute for Physical Chemistry, Department of Chemistry, University of Basel, Basel 4058, Switzerland; Department of Biosystems Science and Engineering, ETH Zurich, Basel 4058, Switzerland; Department of Chemical and Biological Engineering, University of Wisconsin-Madison, Madison, WI 53706, USA; Department of Biomedical Engineering, University of Minnesota, Minneapolis, MN 55455, USA; Program in Mathematical, Computational, and Systems Biology, University of California, Irvine, CA 92697-2280, USA; Center for Synthetic Biology, University of California, Irvine, CA 92697, USA; Department of Biomedical Engineering, University of California, Irvine, CA 92697, USA; Department of Chemical and Biological Engineering, University of Wisconsin-Madison, Madison, WI 53706, USA; Department of Neurological Surgery, University of Wisconsin-Madison, Madison, WI 53706, USA; Department of Biomedical Engineering, University of Minnesota, Minneapolis, MN 55455, USA; Department of Chemical Engineering and Materials Science, University of Minnesota, Minneapolis, MN 55455, USA; Department of Molecular Biology and Biochemistry, University of California, Irvine, CA 92697, USA; Department of Chemistry, University of California, Irvine, CA 92697, USA; Center for Synthetic Biology, University of California, Irvine, CA 92697, USA; Department of Biomedical Engineering, University of California, Irvine, CA 92697, USA; Institute for Physical Chemistry, Department of Chemistry, University of Basel, Basel 4058, Switzerland; Swiss Nanoscience Institute, University of Basel, Basel 4056, Switzerland; Department of Biosystems Science and Engineering, ETH Zurich, Basel 4058, Switzerland

**Keywords:** biotechnology, diagnostics, high-throughput screening, pharmaceuticals, protein engineering, *Saccharomyces cerevisiae*, yeast surface display

## Abstract

Yeast surface display (YSD) is a powerful tool in biotechnology that links genotype to phenotype. In this review, the latest advancements in protein engineering and high-throughput screening based on YSD are covered. The focus is on innovative methods for overcoming challenges in YSD in the context of biotherapeutic drug discovery and diagnostics. Topics ranging from titrating avidity in YSD using transcriptional control to the development of serological diagnostic assays relying on serum biopanning and mitigation of unspecific binding are covered. Screening techniques against nontraditional cellular antigens, such as cell lysates, membrane proteins, and extracellular matrices are summarized and techniques are further delved into for expansion of the chemical repertoire, considering protein–small molecule hybrids and noncanonical amino acid incorporation. Additionally, in vivo gene diversification and continuous evolution in yeast is discussed. Collectively, these techniques enhance the diversity and functionality of engineered proteins isolated via YSD, broadening the scope of applications that can be addressed. The review concludes with future perspectives and potential impact of these advancements on protein engineering. The goal is to provide a focused summary of recent progress in the field.

## 1. Introduction

Proteins play a pivotal role in nearly all biological processes, serving as catalysts, structural components, and signaling molecules. With their remarkable versatility and functional diversity, engineered proteins represent crucial elements in the development of novel therapeutics and diagnostics. The ability to engineer proteins with enhanced or novel functions has significant implications in fields ranging from biotechnology and pharmaceuticals to materials science and renewable energy. Over the years, protein engineering techniques have undergone significant advancements, enabling researchers to explore protein sequence space and optimize protein phenotypes with increasing speed and efficiency.

Among the various protein engineering methodologies, yeast surface display has emerged as a powerful tool, particularly in the context of high-throughput screening. Yeast, especially *Saccharomyces cerevisiae*, has gained prominence due to its eukaryotic nature, ease of genetic manipulation, and the availability of robust display systems. The display of proteins on the yeast cell surface allows for the direct screening of vast protein libraries, enabling a straightforward genotype–phenotype linkage and rapid identification of variants with desired properties. Yeast surface display involves the genetic modification of yeast cells to express a target protein on their surface. The expressed proteins can then be screened for desired traits, such as the ability to bind to a specific molecule, through techniques like fluorescence-activated cell sorting (FACS). This unique ability to link genotype and phenotype makes yeast surface display an indispensable tool in protein engineering.

In recent years, several new methods for protein engineering and high-throughput screening using yeast surface display have been developed. This focused literature review aims to provide an overview of the latest advancements in protein engineering and high-throughput screening through yeast surface display. Specifically, we will highlight methods designed to addresses issues such as a lack of control of multivalency/avidity effects, unspecific binding when panning yeast display libraries against human serum, challenges faced when screening against nonstandard targets (e.g., cell lysates, membrane proteins, and extracellular matrix components), expansion of the chemical repertoire, and finally in vivo gene diversification by continuous evolution. These innovative approaches aim to enhance the diversity and functionality of engineered proteins that can be isolated by yeast surface display, ultimately broadening the scope of protein engineering applications. By compiling and summarizing these novel techniques, this review highlights strategies that have contributed to the recent progress in the field.

## 2. Titrating Avidity in Yeast Display using Transcriptional Control

The most commonly used yeast surface display system, originally developed by Boder and Wittrup in 1997, relies on the a-agglutinin complex formed by the two subunits Aga1p and Aga2p, which are cell adhesion glycoproteins that enhance aggregation of budding yeast cells during mating. The Aga1p subunit contains a glycosyl phosphatidylinositol motif (GPI) that anchors it to glycans on the cell wall. Aga2p is then bound to Aga1p through a pair of disulfide bonds.^[1]^ In YSD, the *AGA1* and *AGA2* genes are controlled by a galactose promoter to synthesize and display proteins of interest (POIs) fused to *AGA2*. One challenge in implementing this system is that differences in sequence, size, and/or structure of Aga2p fusions can generate considerable variation in the expression/display level. This effect can present challenges in certain phenotypic assays to compare clones. Uncontrolled protein copy numbers in display experiments can hinder high-throughput screening/selection methods.^[2]^ The ability to regulate protein display levels can therefore significantly benefit protein engineering efforts by minimizing multivalency effects, for example, when panning against immobilized or insoluble antigens, or when comparing clones in head-to-head phenotypic assays. To address this issue, Wang et al. developed a helper display plasmid that provides two different display levels;^[3]^ Zahradnik et al. constructed plasmids that enable a broad range of intra/extracellular protein expression profiles;^[4]^ and Stern et al. regulated the level of protein display by shaving displayed proteins off the surface of the cells using dithiothreitol reducing agents.^[5]^

**Figure 1.**
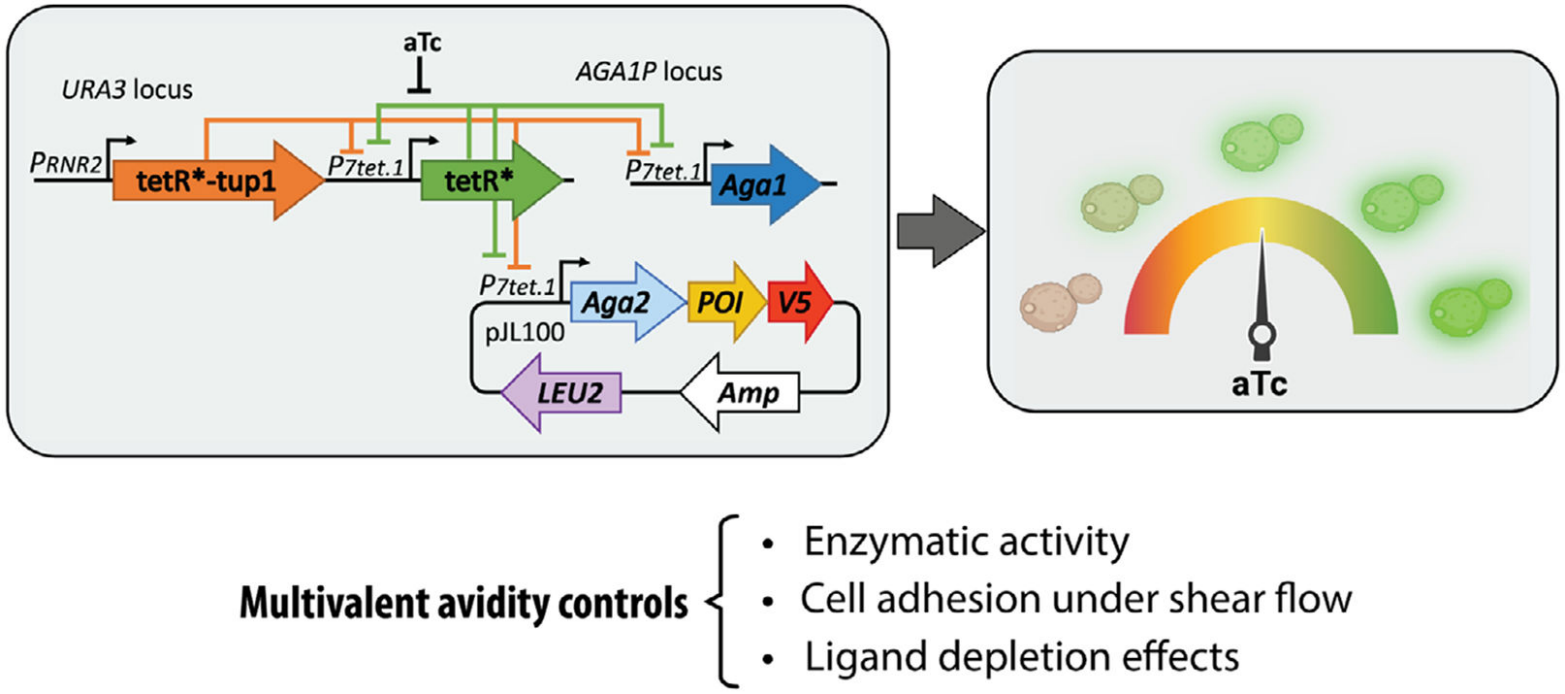
The YTD system regulates the displayed protein density and related phenotypic cellular activities. The YTD platform controls the density of displayed proteins per cell using a titratable gene circuit. The synthetic transcriptional controller provides negative transcriptional feedback to titrate production of Aga2p-fusion proteins dependent on aTc. Control of multivalency (i.e., avidity) influence phenotypic features of the cells displaying the protein of interest, including enzymatic turnover, cell adhesion under flow, and the extent of ligand depletion in yeast-based affinity determination assays. P_7tet.1_, tunable promoter; P_RNR2_, constitutive promoter; tetR, tetracycline repressor; tup1, active yeast repressor; LEU2, auxotrophic gene marker for leucin biosynthesis; V5, epitope tag; Amp: ampicillin.

Recently, we developed a yeast-titratable display (YTD) platform that allows tight control over yeast surface display levels of POIs.^[6]^ The YTD system consists of an engineered yeast strain that genetically modulates the transcription of a genomic copy of *AGA1* and an episomal *AGA2-POI* gene construct. Transcription is modulated through a tetracycline repressor (TetR) negative feedback circuit where TetR represses and controls its own synthesis. Adding anhydrotetracycline (aTc) to the growth medium relieves TetR repression, leading to tunable and precise protein display in an aTc dose-dependent manner (Figure 1). The YTD system displays Aga2p fusions across a range of aTc concentrations from 0 to 200 ng ml^−1^, resulting in maximum levels of the POI displayed per cell and percentage of cells displaying POI at aTc concentrations above ≈60–100 ng ml^−1^, regardless of the POI’s identity. Compared to the standard galactose induction system, the YTD strain displays approximately tenfold less total protein under saturating conditions. It reaches the maximum display levels within 5 h of induction, which is significantly shorter than the time typically required for galactose induction (24–48 h).

We demonstrated the capabilities of the YTD system in different phenotypic assays. First, we showed how controlling the copy number of glucose oxidase enzymes (wild type; M556L; M556L-M561S) displayed on the yeast surface allowed quantitative comparison in catalytic activity of different monoclonal enzyme populations when expressed at equivalent levels. By normalizing the amount of enzyme on the surface of the cells, differences in catalytic activity measured between variants could be attributed directly to the catalytic activity of the assayed enzymes and were in line with previously reported catalytic data for the same variants.^[7]^

We also showed how the YTD system can be used to control adhesion of yeasts bound to a ligand-functionalized coverglass disk and exposed to shear stress.^[8]^ Using a gradient of aTc, yeast populations displaying varying levels of CttA X-module dockerin III (XDocIII) and adhered to a coverglass disk functionalized with a low density of the partner protein Cohesin E (CohE) were exposed to a shear gradient upon rapidly spinning the substrate. After spinning, the remaining cells bound to the surface were imaged, and the sigmoidal distribution of cell density was quantified to identify the shear stress required to detach 50% of the cell population. We found that maximum adhesion was achieved by cells induced at the maximum protein display level (aTc >60 ng ml^−1^) and that reducing the avidity using the YTD system led to a decreased ability to withstand the shear stress, thereby altering the adhesion profiles of the yeast populations induced at different aTc levels through avidity effects.

Finally, we showed how the ability to titrate display levels can be advantageous in miniaturizing affinity-constant measurements while addressing the ligand depletion effect. To avoid overestimating the equilibrium dissociation constant (*K*_D_), we showed how the YTD could downregulate display levels and maintain assay conditions that avoid ligand depletion with reaction volumes suitable for microtiter plates. Comparing *K*_D_ determinations in 100 μL reactions, the standard yeast display system with a high protein level on the surface yielded a *K*_D_ ≈ 24 nM, approximately sevenfold higher than the true value (*K*_D_ = 3.65 nM) due to a ligand depletion artifact. Conversely, the YTD yielded *K*_D_ ≈ 3 nM at low aTc levels (1–5 ng ml^−1^ aTc). The YTD system enables the titration of multivalency and adjustment of the affinity-based screening stringency based on the number of receptors displayed on the surface of the cells.

The YTD system was shown to control phenotypic cell activity by an autorepressible genetic circuit coupled to the agglutinin system architecture. This and other methods for avidity control in yeast surface display are likely to be used more frequently in biomolecular engineering projects in the near future.

## 3. Immunological and Serological Methods using Yeast Display

In vitro diagnostics tests, specifically serological tests, are designed to detect the presence of blood or serum antibodies or antibody-like molecules associated with specific diseases. Common serological assay techniques include enzyme-linked immunosorbent assays (ELISAs), rapid immunochromatographic tests, along with chemiluminescent and electrochemical detection schemes. In all of these techniques, the requirements of recombinant production of protein antigens as bait molecules often pose limitations on the development of serological methods. An early report of the use of yeast display as an immunoassay platform was reported by Guo et al., where an ELISA was established on yeast cells to measure basic model protein and antibody binding.^[9]^ The COVID-19 pandemic further highlighted that serology assays would ideally be scalable for population-level monitoring, as well as highly sensitive, specific, and rapidly adaptable to emerging variants of a pathogen at a low cost per test.

The yeast surface display system is a promising biotechnology platform for the advancement of serology assays. The general approach for on-yeast serology involves transforming yeasts with plasmids encoding the bait protein fused with a yeast mating factor (Aga2), which is displayed on the outer cell wall. By relying on the yeast to generate the antigen bait molecules, it eliminates the necessity for purifying recombinant antigen from host cells. The yeast’s eukaryotic protein quality control system and posttranslational modification machinery facilitate expression and correct folding of many antigens.

In vitro yeast display has been used to study antibody interactions in humans. In recent work, Ring and co-workers developed a novel method for rapid extracellular antigen profiling (REAP) at high throughput using YSD to discover autoantibodies against medically relevant autoimmune targets. By displaying 2688 barcoded human exoproteins on the surface of yeast and panning them against patient serum immunoglobulins, the team identified antigens from the sorted yeast by next-generation sequencing. Several autoantibodies present in autoimmune polyglandular syndrome type 1 (APS-1) and systemic lupus erythematosus (SLE) conditions were newly identified^[10,11]^ in autoimmune patients. The platform was then employed to screen individuals infected with SARS-CoV-2 and elucidated mechanisms of autoantibodies present in COVID-19 infections.^[12]^

**Figure 2.**
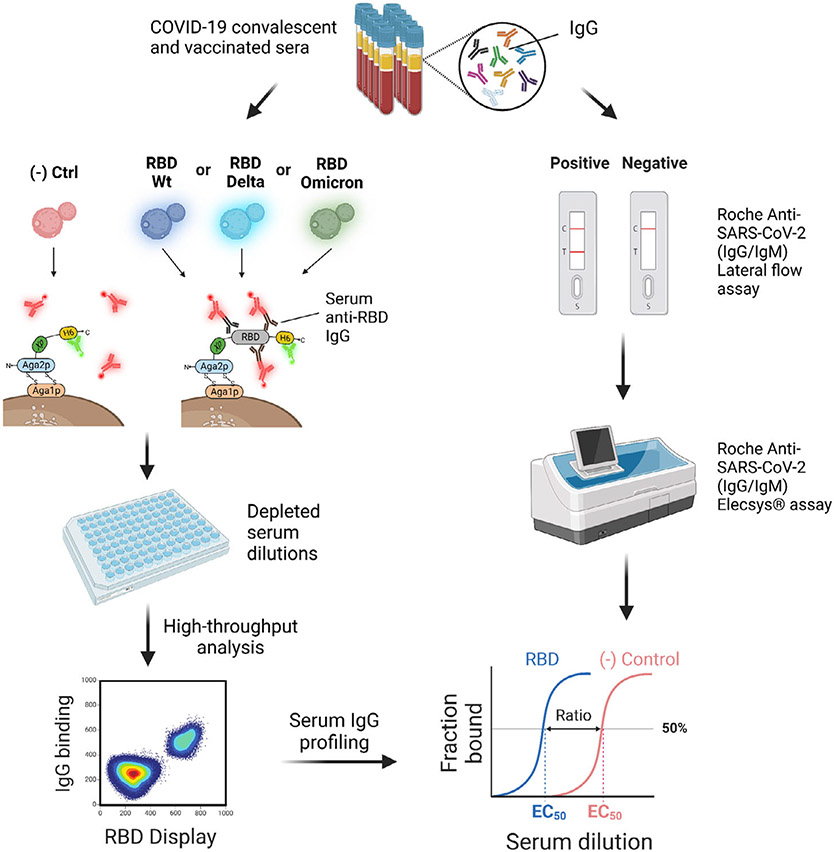
Scheme of the yeast serological immunoassay. Four yeast lines displaying a negative control or one of three SARS-CoV-2 RBD variants are incubated with a dilution series of preprocessed patient sera in multititer plates to titrate binding of RBD-specific human IgGs. One-step immunostaining allows quantifying cells stained double positive for correct display (green fluorescent monoclonal antibodies [mAbs]) and RBD binding (red fluorescent mAbs). Next, high-throughput analytical flow cytometry of the yeast cells quantifies IgG binding to each VOC and compares it to the negative control. Anti-RBD IgGs are profiled as semiquantitative titers for the analyzed sample. The yeast immunoassay was compared to gold-standard methods for qualitatively and quantitatively detecting IgGs, showing strong agreement with both methods. IgG titer distributions can be employed to describe immune responses against a specific VOC at the small population level.

Yeast display is also routinely used to determine the affinity constants of recombinant antibodies,^[13]^ and even serve as whole-cell biosensors of different diseases states.^[14]^ In a recent study, Bloom and co-workers used yeast display to investigate the evolution of SARS-CoV-2 receptor binding domain (RBD) and the propensity of mutations to escape immune recognition by natural and therapeutic antibodies. By displaying an RBD library on the surface of yeast cells and analyzing antibody binding propensity of the variants using deep mutational scanning and next-generation sequencing, they identified several mutations that enhance angiotensin-converting enzyme 2 (ACE2) binding which could then be compared to newly evolving variants. Bloom’s yeast workflow was then optimized to map mutations to the SARS-CoV-2 RBD to demonstrate binding escape of the RBD to different classes of antibodies, and predict the potential emergence of new SARS-CoV-2 variants.^[15-19]^ These techniques coupling yeast display of an antigen library with single-cell sorting and deep sequencing not only provide massive throughput but also yield an immune escape mapping capacity for virtually any pathogen-specific protein that can be displayed on yeast. In related work, Reddy and co-workers used a similar biochemical approach and coupled it with machine learning within highly variable RBD regions to predict future combinatorial variants that could influence ACE2 binding and antibody escape.^[20]^ Recently, Nash and co-workers developed a yeast immunoassay for detecting serum antibodies against the SARS-CoV-2 RBD and associated variants of concern (VOCs) found in Switzerland, namely, Wuhan (B lineage), Delta (B.1.617.2 lineage), and Omicron (B.1.1.529 lineage) RBD (Figure 2).^[21]^ We optimized the assay to minimize ligand depletion and increase the signal-to-noise ratio. Furthermore, we carried out multiplexed serology of anti-SARS-CoV-2 IgG from human sera using four yeast cell lines displaying the VOC in biopanning experiments. The cell lines were challenged with serial dilutions of serum samples from COVID-19 convalescent patients and vaccinated individuals. Seroprofiling of IgG against RBD in human sera achieved 100% specificity and 92% sensitivity compared to the Roche SARS-CoV-2 IgG/IgM rapid antibody lateral flow reference test. Moreover, we compared ten yeast immunoassay titers from vaccinated sera to those obtained with a Roche Elecsys Anti-SARS-CoV-2 immunoassay for the same samples and obtained a high correlation between the methods, pinpointing the semiquantitative determination of IgG titers of the yeast serological assay.

A valuable application of the yeast immunoassay was detecting immune escape against the Omicron variant. The IgG titers acquired from natural immunity after infection were compared against the vaccination-induced IgG titers, resulting in a significant difference between the RBD WT and Delta to the Omicron variant (20- to 100-fold) in convalescent and vaccinated populations.

In general, the yeast serological assay provides information about prior infection and/or vaccination status with SARS-CoV-2 and whether the patient antibodies have altered affinity for a given VOC, offering insights into the immunity of convalescent and vaccinated populations. Finally, we could discern the overall effect of vaccination schemes on IgG titer distributions using the yeast immunoassay. Future developments of yeast serological assays may proceed along a path toward higher sample throughput, for example, by coupling with patient-specific barcodes and sequencing-based readout. A deep sequencing-based output would further enable the inspection of parallel patient-specific immune responses to different pathogen proteins simultaneously at high throughput. As the yeast display component is easily adaptable to new antigens of interest in a resource-efficient way, the yeast immunoassay can be envisioned as a competitive method that will find applications in various viral epidemiology or evolution projects and address current limitations in serological testing.

## 4. Screening Against Nontraditional Cellular Antigens

**Figure 3.**
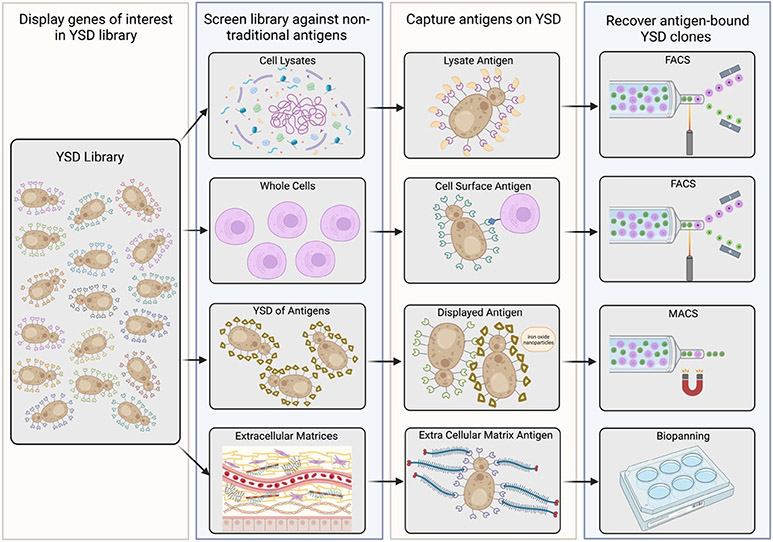
Using nontraditional antigen sources and screens to identify binding proteins. YSD can be leveraged in screens using cell lysates, whole cells, YSD-displayed antigens, and extracellular matrices to identify and engineer binding proteins.

As disease pathologies and cellular targets become further elucidated, novel therapeutic antigens continue to be discovered. Newly discovered antigens from cell or microenvironmental targets are often not soluble proteins for which binding proteins can be identified and engineered using standard yeast and phage display techniques. As a result, screens against such nontraditional cellular antigens using YSD are becoming a more prominent approach to developing new therapeutic binding moieties. Here, we summarize YSD approaches using cell lysates and extracellular matrices as antigen sources for lead binding protein identification and engineering (Figure 3).

### 4.1. YSD with Cell Lysates

YSD libraries are often used to identify therapeutically relevant binding proteins. For such applications, it is beneficial to screen binding protein libraries against targets that are presented in a form that best mimics their native fold and environment. As opposed to recombinant antigen screens, detergent-solubilized cell lysates using nondenaturing detergents can offer the advantage of presenting target antigens in a native or near native form in the background of the complete repertoire of competing antigens. Several studies established the usefulness of cell lysates in YSD antibody screens and affinity maturation.^[22-24]^ This collection of techniques is broadly known as yeast display immunoprecipitation (YDIP), and here we will focus on the most recent adaptations of these methods.

Recent work demonstrated the utility of YDIP screening using an in vivo-sourced cell lysate antigen pool which holds the promise of increased in vivo relevance of the output binding pool.^[25]^ A YSD library of variable lymphocyte receptors (VLRs) was generated from lamprey that were immunized with brain endothelial cell plasma membranes directly fractioned from mouse brain microvessels.^[25]^ To enrich the library for brain endothelial cell membrane targeted VLRs, YDIP was performed using an antigen pool comprising detergent-solubilized, biotinylated lysates of the same mouse brain microvessel plasma membranes that were used for lamprey immunization.^[25]^ YDIP with one round of magnetic activated cell sorting (MACS) followed by one round of FACS led to a tenfold enrichment in yeast-displayed VLRs capable of binding brain microvessel plasma membrane compared to the starting library.^[25]^ Subsequent selection of the library for VLRs that could bind extracellular epitopes was performed by biopanning against mouse brain endothelial cell cultures.^[25]^ While biopanning for enrichment of VLRs binding to extracellular targets was performed using cell cultured substrates rather than in vivo sourced cell lysates, the initial enrichment of the library using the brain endothelial plasma membranes ensured that the resulting pool of VLRs had many variants (16 of 26 clones tested) that could bind relevant antigens in brain microvessels. Therefore, the naturally occurring protein expression profiles in lysates can offer a more physiologically relevant array of therapeutic targets for YSD screens of challenging cell types.

Another recent study directly compared YSD screens using a recombinant antigen versus cell lysate.^[26]^ A YSD library was generated using paired single-chain variable fragments (scFvs) from immunization of the Trianni humanized mouse with recombinant OX40.^[26]^ The subsequent YSD library was screened by flow cytometry with either recombinant OX40 or OX40-containing cell lysate.^[26]^ The scFvs identified as positive OX40 binders were reformatted as mAbs for subsequent binding characterization to assess therapeutic potential. The mAbs produced from scFvs identified in the cell lysate screen all bound cell surface OX40 in a cell-based binding assay.^[26]^ However, of the scFvs identified in the recombinant OX40 screen, only 39% of the reformatted mAbs bound cell surface OX40 in the same cell-based binding assay.^[26]^ The 61% false-positive rate identified in the recombinant OX40 screen was attributed to the monomeric form of OX40 that differs from physiological OX40 which exists as a trimer.^[26]^ Multimeric differences between recombinant and native protein states for certain antigens further support the benefits for lysate screening.

While lysate-sourced antigens have proven useful for many YSD library screens, lysates are not suitable for all antigens as demonstrated in another recent study.^[27]^ Mutagenic libraries of a fibronectin domain and an affibody were expressed in YSD and screened for binders to CD276 or Thy1.^[27]^ Screen workflows for the YSD libraries included an initial step of magnetic bead sorting with recombinant extracellular domains followed by 1) FACS with recombinant extracellular domains, 2) FACS with detergent solubilized cell lysate, or 3) cell biopanning.^[27]^ For the CD276 antigen, FACS with recombinant extracellular domains did not produce fibronectin or affibody binders.^[27]^ Using CD276-containing cellular lysates also did not enrich the libraries for specific binders.^[27]^ However, when cell panning screens were included, CD276-binding proteins in both the fibronectin and affibody YSD libraries were identified.^[27]^ A possible explanation for not observing lysate enrichment in this instance is that the initial FACS enrichment against recombinant epitopes does not share the same protein conformations as epitopes found in the lysate, as was observed by the OX40 study. In instances when lysate enrichment is minimal, it is possible to augment YSD libraries through lysate depletion strategies if an appropriate cell line is available. Previous work showed that cell lines absent of the library target can be used in lysate form as a polyspecificity reagent for library depletion and to measure the cross-reactivity of candidate clones discovered in lysate enrichment screens.^[28]^ Such studies demonstrate the need for employing a multipronged approach to screening YSD libraries depending on the desired therapeutic target.

### 4.2. Membrane Proteins

Membrane proteins are a particularly challenging set of targets for the isolation of binders as they are poorly soluble and recombinant ectodomains are commonly misfolded or do not result in productive identification campaigns for binding proteins. As noted in the previous section, one strategy is to deploy cell lysates in combination with YSD screening. To further address the challenges presented by membrane protein targets, recent studies have provided alternate methods for screening YSD libraries specifically toward membrane protein targets.

One such study using YDIP leveraged the fact that detergent-solubilized complexes can preserve protein–protein interactions much like classic immunoprecipitation procedures. Hence, one can screen for binding protein interactions with cell surface receptors that are, in turn, associated with key cellular machinery or processes. For example, such “functional YDIP” (fYDIP) was used to enrich for binders to blood–brain barrier membrane protein complexes involved in endocytocytic processes.^[29]^ In this study, brain microvessels were isolated from bovine or rat brains and the membrane protein fraction biotinylated.^[29]^ First, a YSD library of nonimmune scFvs was screened for a pool of binders to the detergent-solubilized, biotinylated membrane proteins using MACS.^[29]^ The second round screen employed a FACS protocol to isolate scFv that bound to biotinylated membrane protein complexes that also contained AP-2, a key adaptor protein in clathrin-mediated endocytosis.^[29]^ A total of 31 unique clones were identified from the fYDIP screen. Of the 31, 26 were confirmed to bind proteins in detergent-solubilized blood–brain barrier membrane protein preparations, and 22 of the binding scFvs were confirmed to bind a membrane protein complex that contained AP-2.^[29]^ For validation, ten of the clones were produced as soluble scFv-Fc fusions, and all ten clones demonstrated positive staining of blood–brain barrier membranes in rat brain tissue sections, indicating the in vivo relevance of such a screen.^[29]^

G protein-coupled receptors (GPCRs) are another class of membrane proteins that are notoriously difficult to process through binding protein identification campaigns.^[30]^ A recent study deployed a variation of YSD biopanning where yeasts displaying a mutagenic antibody library were enriched for antibodies with improved binding to the mu opioid GPCR.^[30]^ In a bit more detail, Mu opioid GPCRs were overexpressed in mammalian cells that were then incubated with the mutagenic YSD antibody library.^[30]^ Conjugates of yeast (green reporter) and a mu opioid expressing mammalian cell (red reporter) conjugates were then isolated by dual-color FACS.^[30]^ Following four rounds of selection for the highest YSD binding population, 30 clones were chosen for binding the mu opioid enriched cells.^[30]^ Two lead clones were shown to bind the GPCR with affinities of 76 nM and 280 nM.^[30]^ Subsequently, a kinetic dissociation competition screen using an excess of mu opioid receptor expressing cells (yellow) followed by FACS to identify the persisting yeast (green)-mammalian cell (red) conjugates was successful in further maturing the antibody affinity. The results from the cell-based kinetic screen were similar to those when a traditional YSD kinetic dissociation screen was performed with mu opioid receptor ectodomain.

Another approach for screening YSD libraries for membrane protein binders was developed by using YSD for presentation of both the binder library and the cognate membrane protein antigen.^[31]^ In this case, the membrane proteins, ectodomains, or fragments are co-expressed in YSD format along with an iron oxide-binding protein.^[31]^ When this population of yeast is incubated with iron-oxide nanoparticles, the yeast cells are magnetized for facile magnetic sorting.^[31]^ The binder library YSD pool is then contacted with the target protein-expressing yeast population, and binder-target YSD complexes isolated by MACS. The method was validated using two targets, the cytosolic domain of the mitochondrial membrane protein TOM22 and the extracellular domain of the c-Kit receptor.^[31]^ YSD libraries based on the Sso7d binding scaffold and nanobody scaffolds were screened against the target expressing yeast resulting in lead clones binding their respective membrane proteins on the order of nanomolar affinity.^[31]^ Taken together, difficult targets like membrane proteins can be amenable to YSD techniques when the antigens are presented as detergent-solubilized lysates, overexpressing cell lines or if they themselves are expressed in YSD formats.

### 4.3. Extracellular Matrices

In addition to cell lysates or membrane proteins, the extracellular matrix (ECM) is a growing therapeutic target that can be explored using YSD screens. Target cells, including tumor cells, secrete ECM proteins that can make for highly specific drug delivery targets. Several studies have demonstrated the advantage of therapeutic targeting of the ECM.^[32,33]^ Thus, using YSD libraries to identify and engineer binders to ECM offers another application of YSD.

For example, YSD has been used to identify binders to brain ECM for the targeting of glioblastoma tumors.^[34]^ In this study, a VLR library was generated from lamprey immunized with murine brain microvessel plasma membranes and their associated brain ECM.^[34]^ The resultant YSD library was enriched by two rounds of biopanning on decellularized mouse brain endothelial cell ECM.^[34]^ Following the library enrichment, 285 VLR clones from the YSD library were screened using a high throughput method where candidate VLRs were released from the surface of individual YSD clones and subjected to a comparative binding ELISA against brain ECM or fibroblast ECM. Ten of the 285 clones showed a minimum 2.5-fold preference for brain ECM binding.^[34]^ The lead clone for selective binding to brain ECM, P1C10, was produced and demonstrated selective binding at the site of pathologically exposed brain ECM in mouse glioblastoma models.^[34]^ When conjugated to chemotherapy, P1C10 resulted in increased survival in murine glioblastoma survival studies.^[34]^ Another study demonstrated the utility of using YSD to identify improved nanobodies that target ECM for tumor therapy.^[35]^ In this study, soluble, biotinylated EIIIB domain of fibronectin, a known tumor-specific ECM protein, was used as the antigen to enrich a YSD library of mutagenic nanobodies using standard YSD protocols.^[35]^ The resultant library produced nanobody variants with picomolar affinity for EIIIB.^[35]^ The high affinity nanobody clones were conjugated to immunocytokines and intratumoral administration resulted in improved survival in murine tumor models.^[35]^

## 5. Expansion of the Chemical Repertoire

**Figure 4.**
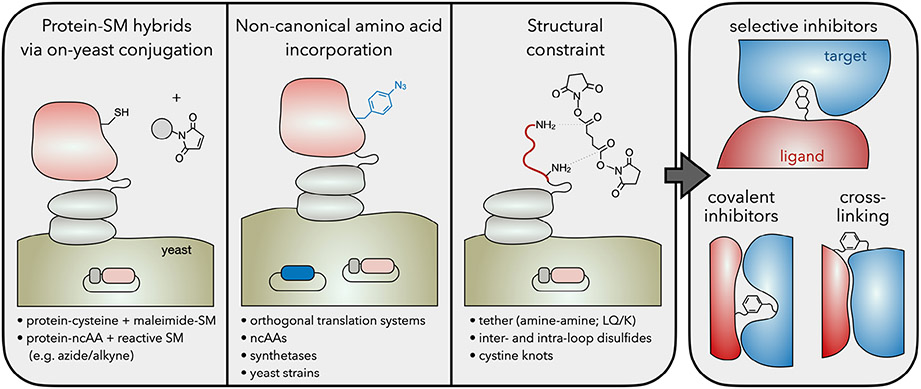
Leveraging chemical biology to expand the chemical repertoire. (Left) Approaches to expand the chemical scope of YSD. SM, small molecule; ncAA, noncanonical amino acid. (Right) These techniques can provide engineered proteins with unique properties such as selective or covalent inhibition or chemical crosss-linking.

Engineered proteins empower molecular targeting and modulation of biological function for therapeutics, diagnostics, industrial biotechnology, and fundamental science. The diversity of sizes, topologies, and chemistries enable a broad array of functionality. Nevertheless, there are numerous chemistries lacking in the 20 canonical amino acids and an array of conformations not accessible by natural polypeptide backbones. Expanded chemical modalities unlock functional mechanisms that are otherwise challenging or impossible to achieve with proteins alone. Multiple molecular engineering strategies have been employed to expand the chemical repertoire while still leveraging the benefits of protein engineering, including genotype-phenotype linkage for high-throughput evaluation and selection (Figure 4).

### 5.1. Protein–Small Molecule Hybrids via On-Yeast Conjugation

Protein–small molecule hybrids have demonstrated a powerful expansion of protein molecular space to improve selective inhibition of kinases^[36-38]^ and carbonic anhydrases^[39]^ via covalent attachment of a nonspecific small molecule pharmacophore to a polypeptide or protein. These studies highlight the utility of proteins with noncanonical chemistries to combine the potency and unique modality of small molecule inhibitors with the selectivity and affinity of proteins while motivating an efficient search of the broad hybrid chemical space. Display technologies have been leveraged to facilitate hybrid engineering using multiple site-specific chemistries and different display methods. Li and Roberts performed mRNA display of a peptide with a single cysteine, conjugated the bromoacetyl derivative of penicillin, and performed binding selections.^[40]^ The resultant peptide–pharmacophore hybrid exhibited stronger potency than peptide or penicillin alone. Phage display of peptides with two cysteines followed by simultaneous cyclization and appendage of a pharmacophore via dihaloketone derivatives has been used by several labs to engineer cyclic peptide–pharmacophore hybrids with strong potency and selectivity.^[41-43]^ Conjugation to oxidized N-terminal serine of phage displayed peptides has also been used.^[44,45]^ The eukaryotic translational machinery^[46]^ and quantitative precision^[47]^ of flow cytometric sorting makes YSD an attractive solution for engineering more complex protein-based hybrid ligands. The Hackel lab yeast-displayed fibronectin protein domains with a sole cysteine, performed site-specific conjugation with maleimide-pharmacophore, and identified potent, selective inhibitors of carbonic anhydrase isoforms via flow cytometric sorting.^[48]^ The protein–pharmacophore hybrids exhibited improved potency and selectivity relative to protein or small molecule alone. Several of the engineered hybrids were reformatted by the Van Deventer lab via replacement of cysteine by azide- or alkyne-containing ncAAs and using click chemistry to tether a set of sulfonamide variants.^[49]^ Multiple novel chemistries and pharmacophores enabled strong activity.

### 5.2. ncAA Incorporation

The incorporation of ncAAs into displayed proteins and peptides unlocks novel chemical modalities and enables true site-selective conjugation. Other display technologies,^[50,51]^ including mRNA,^[52-55]^ phage,^[56,57]^ and bacterial^[58]^ display, were initially used to engineer ncAA-containing proteins. Van Deventer has pioneered ncAA incorporation and conjugation in yeast display. Initially, the concept was validated as antibody fragments were yeast displayed with incorporation of azido- and acetyl-phenylalanine ncAAs via amber suppression and conjugated via copper-catalyzed azide-alkyne cycloaddition.^[59]^ A series of studies optimized elements of the platform. Performant orthogonal translation systems were then identified for an array of ncAAs.^[60]^ The orthogonal translation system was integrated along with the yeast display gene constructs onto a single plasmid to simplify genetic modulation of yeast.^[61]^ The *Methanomethylophilus alvus* pyrollysyl tRNA synthetase was used to incorporate multiple ncAAs, particularly N_ε_-Boc-lysine.^[62]^ Libraries of tRNA synthetase mutants were sorted to identify variants that incorporate an array of ncAAs including boronphenylalanine and 3,4-dihydroxy-phenylalanine.^[63]^ A knockout screen of the yeast genome identified two single-gene deletions that improved ncAA incorporation.^[64]^ In addition to the aforementioned application to fibronectin–pharmacophore hybrids, Van Deventer and team yeast displayed engineered antibody fragments with a ncAA-incorporated adjacent to the binding site; screening across multiple sites and ncAAs yielded multiple nM binders; click chemistry conjugation resulted in maintained binding in several cases and empowered photoactivated antibody-target cross-linking.^[65]^ This concept was extended via incorporation of photoreactive (azido-phenylalanine) and proximity-reactive (O-(2-bromoethyl)-tyrosine) ncAAs adjacent to the binding site in single-domain antibodies to create covalent inhibitors of botulinum neurotoxin light chain A.^[66]^

The utility of ncAAs reaches beyond engineered ligands. Expansion of the chemical repertoire of enzymes can enable catalysis of otherwise intractable chemical reactions.^[67]^ A protein bearing a promiscuous binding pocket was converted into an enzyme via incorporation of a catalytic *p*-aminophenylalanine residue.^[68]^ The Green lab has engineered novel hydrolytic enzymes^[69]^ and [2 + 2] cycloaddition photoenzymes^[70]^ with noncanonical catalytic residues. A future opportunity will be to merge the enzyme engineering capabilities of YSD^[71]^ with ncAA incorporation.

### 5.3. Structural Constraint

YSD techniques have been used to expand the structural repertoire via cyclic constraint. The Rao lab has created cyclic peptides on the yeast surface by displaying a linear peptide and crosslinking via two approaches: chemically tethering the free N-terminal amine and the tenth-residue lysine amine with a disuccinimidyl glutarate crosslinker;^[31]^ and enzymatically tethering an N-terminal LQ to an 11th-residue lysine via transglutaminase.^[72]^ YSD has also been used for its ability to form inter- and intraloop disulfide bonds in alternative scaffolds.^[73-75]^ Furthering the ability to form proper disulfide bonds, the Cochran lab has leveraged the eukaryotic folding capabilities of yeast to engineer cystine knot peptides.^[76]^ YSD selections of combinatorial libraries of knottins have enabled engineering of high-affinity, selective binders to multiple integrins, which have been used in molecular imaging and therapeutics.^[77-80]^

## 6. Continuous Evolution in Yeast

Continuous evolution relies on in vivo gene diversification strategies, and allows evolution to proceed in a more natural way, where genetic sequences are spontaneously mutated and the host is subjected to selective or screening pressure. OrthoRep is a system for in vivo continuous directed evolution in yeast, consisting of a linear plasmid (p1) that is replicated by its own orthogonal DNA polymerase (terminal protein DNA polymerase 1 [TP-DNAP1]) that does not replicate the host genome. When a gene encoding a protein of interest is placed on p1, it is exclusively propagated through the action of TP-DNAP1. TP-DNAP1 has been engineered to introduce errors at a rate ≈100 000-fold higher than the mutation rate of genomic replication.^[81]^ This enables the rapid in vivo diversification of any gene encoded on p1 without adversely affecting genomic DNA replication. Through passaging of yeast under the appropriate selective pressure, directed evolution of the protein of interest toward new functions can be achieved with minimal researcher intervention.^[82-84]^

**Figure 5.**
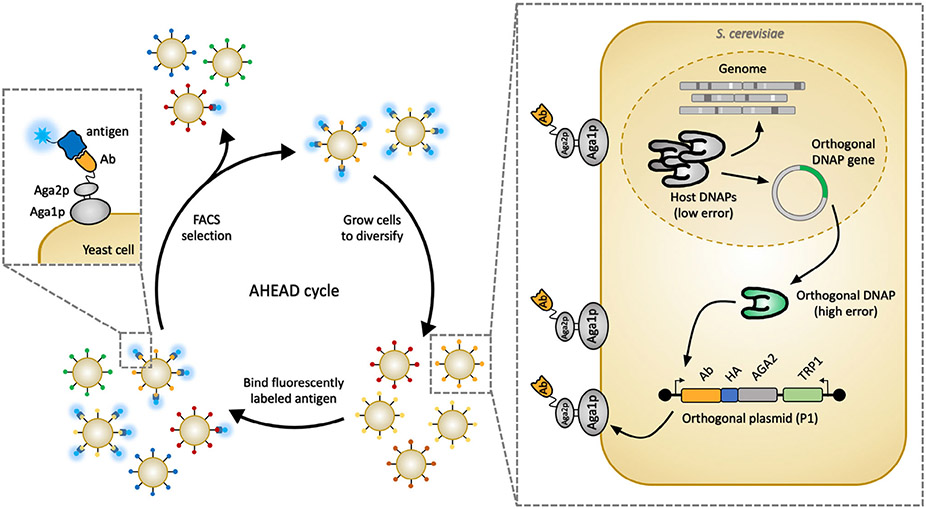
AHEAD. AHEAD^[85]^ pairs OrthoRep^[81,86]^ with yeast surface display for a platform that enables the rapid discovery of high-affinity specific antibodies.

In all directed evolution campaigns, appropriate methods of selection for the desired function are paramount to success. To harness the capabilities of the OrthoRep system for the rapid directed evolution of antibodies and other binding proteins, the OrthoRep system was paired with YSD resulting in the AHEAD (Autonomous Hypermutation yEast surfAce Display) platform (Figure 5). In AHEAD, the coding sequence of a binding protein such as an antibody fragment is encoded on p1 as a fusion to the agglutinin, Aga2, alongside a tag for detecting surface display. Aga1 is expressed from the genome at its native locus under the inducible GAL promoter. Upon galactose induction, the antibody fragment is displayed on the yeast surface through the Aga1–Aga2 yeast display architecture first described by Boder and Wittrup.^[1]^ As a result of the high mutation rate of the orthogonal DNAP propagating p1, mutations are autonomously and rapidly introduced in the antibody fragment gene. After a few cell divisions, a large population of cells displaying diverse antibodies are produced, allowing for the sampling of antibodies with increased binding affinity to target antigens. The cells are stained with fluorescently labeled antigen and sorted by FACS to select variants that have accumulated mutations beneficial to binding of the antigen. After sorting, cells are grown to saturation, allowing for the accumulation of more mutations. Repeated rounds of growth and sorting enable the rapid evolution (i.e., affinity maturation) of strongly binding and highly specific antibody fragments.

AHEAD has been used to obtain nanomolar nanobodies in as little as 2 weeks.^[85]^ Traditional methods of antibody generation, predominantly animal immunization, suffer setbacks including high cost, low throughput, and incompatibility with self-antigens or those requiring solubilization. AHEAD offers a rapid and parallelizable in vitro route to highly potent antibodies compared to animal immunization or traditional in vitro selection methods involving enrichment of binders from large libraries of nonmutating antibody fragment sequences. AHEAD’s use in the rapid and parallel evolution of nanobodies targeting different epitopes of the SARS-CoV-2 spike protein provides a demonstration of the system’s usefulness in creating antibody cocktails that could minimize the likelihood of viral escape in future infectious disease applications. AHEAD also permits the selection of antibodies against user-specified epitopes of an antigen, which can be accomplished by counter-selecting against binders of undesirable epitopes during AHEAD cycles. Recently, several naïve antibody libraries have been built into the AHEAD system, allowing for the selection of an initial binder followed by its affinity maturation into a strong binder all in one end-to-end process.

## 7. Conclusions

Yeast surface display has emerged as a powerful tool for high-throughput screening, particularly due to the advantages offered by yeast as a host organism. The ability to display proteins on the yeast cell surface enables direct screening of large protein libraries, facilitating rapid identification of variants with desired properties. The development of new methods and techniques in this field promises to accelerate the development of therapeutics and diagnostics by allowing the rapid creation and phenotypic validation of proteins with enhanced or novel functions. With this review, we provided an overview of the latest methods and techniques in this field. We highlighted the development of innovative approaches to address challenges such as multivalency/avidity effects, screening and serology against human serum, screening against nonstandard targets, expanding the chemical repertoire, and in vivo gene diversification through continuous evolution. Looking ahead, further advancements in yeast surface display and protein engineering are anticipated. Several continual and emerging technology trends are expected to work synergistically with yeast surface display in coming years. These trends include advances in artificial intelligence and machine learning, protein structure design, advanced automation, and decreasing cost of oligonucleotide synthesis and next-generation DNA sequencing. Additionally, the application of yeast surface display in more diverse areas, such as environmental remediation and personalized medicine, may open up new opportunities. Yeast surface display has proven to be a valuable platform for protein engineering and high-throughput screening, and ongoing advancements in this field will undoubtedly contribute to innovation across multiple scientific disciplines.

## References

[1] Boder ET, Wittrup KD, Nat. Biotechnol 1997, 15, 553. 9181578 10.1038/nbt0697-553

[2] Teymennet-Ramírez KV, Martínez-Morales F, Trejo-Hernández MR, Front. Bioeng. Biotechnol 2021, 9, 794742. 35083204 10.3389/fbioe.2021.794742 PMC8784408

[3] Wang KC, Patel CA, Wang J, Wang J, Wang X, Luo PP, Zhong P, J. Immunol. Methods 2010, 354, 11. 20097202 10.1016/j.jim.2010.01.006

[4] Zahradník J, Dey D, Marciano S, Kolářová L, Charendoff CI, Subtil A, Schreiber G, ACS Synth. Biol 2021, 10, 3445. 34809429 10.1021/acssynbio.1c00395 PMC8689690

[5] Stern LA, Csizmar CM, Woldring DR, Wagner CR, Hackel BJ, ACS Comb. Sci 2017, 19, 315. 28322543 10.1021/acscombsci.6b00191 PMC5521271

[6] Lopez-Morales J, Vanella R, Kovacevic G, Santos MS, Nash MA, ACS Synth. Biol 2023, 12, 419. 36728831 10.1021/acssynbio.2c00351 PMC9942200

[7] Kovačević G, Ostafe R, Fischer R, Prodanović R, Biochem. Eng. J 2019, 146, 143.

[8] Santos MS, Liu H, Schittny V, Vanella R, Nash MA, Biophys. Rep 2022, 2, 100035. 10.1016/j.bpr.2021.100035 PMC8904261 35284851

[9] Guo Y, Cheng D, Lee TY, Wang J, Hsing I-M, Anal. Chem 2010, 82, 9601. 21067137 10.1021/ac102241k

[10] Wang EY, Dai Y, Rosen CE, Schmitt MM, Dong MX, Ferré EMN, Liu F, Yang Y, González-Hernández JA, Meffre E, Hinchcliff M, Koumpouras F, Lionakis MS, Ring AM, Cell Rep. Methods 2022, 2, 100172. 35360706 10.1016/j.crmeth.2022.100172 PMC8967185

[11] Cheng D, Guo Y, Hsing I-M, Analyst 2012, 137, 999. 22193284 10.1039/c2an15946e

[12] Wang EY, Mao T, Klein J, Dai Y, Huck JD, Jaycox JR, Liu F, Zhou T, Israelow B, Wong P, Coppi A, Lucas C, Silva J, Oh JE, Song E, Perotti ES, Zheng NS, Fischer S, Campbell M, Fournier JB, Wyllie AL, Vogels CBF, Ott IM, Kalinich CC, Petrone ME, Watkins AE, Yale IMPACT Team, Dela Cruz C, Farhadian SF, Schulz WL, Ma S, , Nature 2021, 595, 283. 34010947 10.1038/s41586-021-03631-y PMC13130511

[13] Chao G, Lau WL, Hackel BJ, Sazinsky SL, Lippow SM, Wittrup KD, Nat. Protoc 2006, 1, 755. 17406305 10.1038/nprot.2006.94

[14] Park M, Sensors 2020, 20, 2775.32414189

[15] Starr TN, Greaney AJ, Hilton SK, Ellis D, Crawford KHD, Dingens AS, Navarro MJ, Bowen JE, Tortorici MA, Walls AC, King NP, Veesler D, Bloom JD, Cell 2020, 182, 1295. 32841599 10.1016/j.cell.2020.08.012 PMC7418704

[16] Greaney AJ, Starr TN, Barnes CO, Weisblum Y, Schmidt F, Caskey M, Gaebler C, Cho A, Agudelo M, Finkin S, Wang Z, Poston D, Muecksch F, Hatziioannou T, Bieniasz PD, Robbiani DF, Nussenzweig MC, Bjorkman PJ, Bloom JD, Nat. Commun 2021, 12, 4196. 34234131 10.1038/s41467-021-24435-8 PMC8263750

[17] Greaney AJ, Loes AN, Crawford KHD, Starr TN, Malone KD, Chu HY, Bloom JD, Cell Host. Microbe 2021, 29, 463. 33592168 10.1016/j.chom.2021.02.003 PMC7869748

[18] Starr TN, Greaney AJ, Hannon WW, Loes AN, Hauser K, Dillen JR, Ferri E, Farrell AG, Dadonaite B, McCallum M, Matreyek KA, Corti D, Veesler D, Snell G, Bloom JD, Science 2022, 377, eabo7896. 35762884 10.1126/science.abo7896 PMC9273037

[19] Starr TN, Greaney AJ, Addetia A, Hannon WW, Choudhary MC, Dingens AS, Li JZ, Bloom JD, Science 2021, 371, 850. 33495308 10.1126/science.abf9302 PMC7963219

[20] Taft JM, Weber CR, Gao B, Ehling RA, Han J, Frei L, Metcalfe SW, Overath MD, Yermanos A, Kelton W, Reddy ST, Cell 2022, 185, 4008. 36150393 10.1016/j.cell.2022.08.024 PMC9428596

[21] Lopez-Morales J, Vanella R, Utzinger T, Schittny V, Hirsiger J, Osthoff M, Berger C, Guri Y, Nash MA, iScience 2023, 26, 106648. 37124419 10.1016/j.isci.2023.106648 PMC10089669

[22] Tillotson BJ, Cho YK, Shusta EV, Methods 2013, 60, 27. 22449570 10.1016/j.ymeth.2012.03.010 PMC3405166

[23] Cho YK, Shusta EV, Protein Eng. Des. Sel 2010, 23, 567. 20498037 10.1093/protein/gzq029 PMC2920304

[24] Cho YK, Chen I, Wei X, Li L, Shusta EV, J. Immunol. Methods 2009, 341, 117. 19041873 10.1016/j.jim.2008.11.005 PMC2637942

[25] Lajoie JM, Katt ME, Waters EA, Herrin BR, Shusta EV, Sci. Rep 2022, 12, 6044. 35411012 10.1038/s41598-022-09962-8 PMC9001667

[26] Medina-Cucurella AV, Mizrahi RA, Asensio MA, Edgar RC, Leong J, Leong R, Lim YW, Nelson A, Niedecken AR, Simons JF, Spindler MJ, Stadtmiller K, Wayham N, Adler AS, Johnson DS, Antibodies 2019, 8, 17. 31544823 10.3390/antib8010017 PMC6640694

[27] Stern LA, Lown PS, Kobe AC, Abou-Elkacem L, Willmann JK, Hackel BJ, ACS Comb. Sci 2019, 21, 207. 30620189 10.1021/acscombsci.8b00156 PMC6411437

[28] Xu Y, Roach W, Sun T, Jain T, Prinz B, Yu T-Y, Torrey J, Thomas J, Bobrowicz P, Vásquez M, Wittrup KD, Krauland E, Protein Eng. Des. Sel 2013, 26, 663. 24046438 10.1093/protein/gzt047

[29] Lajoie JM, Cho YK, Frost D, Bremner S, Li L, Shusta EV, Protein Eng. Des. Sel 2019, 32, 219. 31769480 10.1093/protein/gzz035 PMC7017056

[30] Yang Z, Wan Y, Tao P, Qiang M, Dong X, Lin C-W, Yang G, Zheng T, Lerner Proc RA. Natl. Acad. Sci. U. S. A 2019, 116, 14971. 10.1073/pnas.1908571116 PMC6660777 31285332

[31] Bacon K, Burroughs M, Blain A, Menegatti S, Rao BM, ACS Comb. Sci 2019, 21, 817. 31693340 10.1021/acscombsci.9b00147

[32] Woodworth GF, Dunn GP, Nance EA, Hanes J, Brem H, Front. Oncol 2014, 4, 126. 25101239 10.3389/fonc.2014.00126 PMC4104487

[33] Lockman PR, Mittapalli RK, Taskar KS, Rudraraju V, Gril B, Bohn KA, Adkins CE, Roberts A, Thorsheim HR, Gaasch JA, Huang S, Palmieri D, Steeg PS, Smith QR, Clin. Cancer Res 2010, 16, 5664. 20829328 10.1158/1078-0432.CCR-10-1564 PMC2999649

[34] Umlauf BJ, Clark PA, Lajoie JM, Georgieva JV, Bremner S, Herrin BR, Kuo JS, Shusta EV, Sci. Adv 2019, 5, eaau4245. 31106264 10.1126/sciadv.aau4245 PMC6520025

[35] Lutz EA, Jailkhani N, Momin N, Huang Y, Sheen A, Kang BH, Wittrup KD, Hynes RO, PNAS Nexus 2022, 1, gac244. 10.1093/pnasnexus/pgac244 PMC9802395 36712341

[36] Gower CM, Thomas JR, Harrington E, Murphy J, Chang MEK, Cornella-Taracido I, Jain RK, Schirle M, Maly DJ, ACS Chem. Biol 2016, 11, 121. 26505072 10.1021/acschembio.5b00847 PMC4943861

[37] Schneider TL, Mathew RS, Rice KP, Tamaki K, Wood JL, Schepartz A, Org. Lett 2005, 7, 1695. 15844883 10.1021/ol050179o

[38] Meyer SC, Shomin CD, Gaj T, Ghosh I, J. Am. Chem. Soc 2007, 129, 13812. 17944472 10.1021/ja076197d

[39] Andersson T, Lundquist M, Dolphin GT, Enander K, Jonsson B-H, Nilsson JW, Baltzer L, Chem. Biol 2005, 12, 1245. 16298304 10.1016/j.chembiol.2005.08.018

[40] Li S, Roberts RW, Chem. Biol 2003, 10, 233. 12670537 10.1016/s1074-5521(03)00047-4

[41] Chen S, Lovell S, Lee S, Fellner M, Mace PD, Bogyo M, Nat. Biotechnol 2021, 39, 490. 33199876 10.1038/s41587-020-0733-7 PMC8043995

[42] Ekanayake AI, Sobze L, Kelich P, Youk J, Bennett NJ, Mukherjee R, Bhardwaj A, Wuest F, Vukovic L, Derda R, J. Am. Chem. Soc 2021, 143, 5497. 33784084 10.1021/jacs.1c01186

[43] Zheng M, Chen F-J, Li K, Reja RM, Haeffner F, Gao J, J. Am. Chem. Soc 2022, 144, 15885. 35976695 10.1021/jacs.2c07375 PMC9440474

[44] Kugler M, Hadzima M, Dzijak R, Rampmaier R, Srb P, Vrzal L, Voburka Z, Majer P, Řezáčová P, Vrabel M, RSC Med. Chem 2023, 14, 144. 36760748 10.1039/d2md00330a PMC9890587

[45] Ng S, Lin E, Kitov PI, Tjhung KF, Gerlits OO, Deng L, Kasper B, Sood A, Paschal BM, Zhang P, Ling C-C, Klassen JS, Noren CJ, Mahal LK, Woods RJ, Coates L, Derda R, J. Am. Chem. Soc 2015, 137, 5248. 25860443 10.1021/ja511237n PMC5553193

[46] Sun Z, Brodsky JL, J. Cell Biol 2019, 218, 3171. 31537714 10.1083/jcb.201906047 PMC6781448

[47] VanAntwerp JJ, Wittrup KD, Biotechnol. Prog 2000, 16, 31. 10662486 10.1021/bp990133s

[48] Lewis AK, Harthorn A, Johnson SM, Lobb RR, Hackel BJ, Cell Chem. Biol 2022, 29, 328. 34363759 10.1016/j.chembiol.2021.07.013 PMC8807807

[49] Huang M, Rueda-Garcia M, Harthorn A, Hackel BJ, Van Deventer JA, bioRxiv 2023, 10.1101/2023.05.12.540568. PMC11146673 38230650

[50] Passioura T, Katoh T, Goto Y, Suga H, Annu. Rev. Biochem 2014, 83, 727. 24580641 10.1146/annurev-biochem-060713-035456

[51] Iskandar SE, Haberman VA, Bowers AA, ACS Comb. Sci 2020, 22, 712. 33167616 10.1021/acscombsci.0c00179 PMC8284915

[52] Josephson K, Hartman MCT, Szostak JW, J. Am. Chem. Soc 2005, 127, 11727. 16104750 10.1021/ja0515809

[53] Hayashi Y, Morimoto J, Suga H, ACS Chem. Biol 2012, 7, 607. 22273180 10.1021/cb200388k

[54] Horiya S, Bailey JK, Temme JS, Guillen Schlippe YV, Krauss IJ, J. Am. Chem. Soc 2014, 136, 5407. 24645849 10.1021/ja500678v PMC4004241

[55] Passioura T, Liu W, Dunkelmann D, Higuchi T, Suga H, J. Am. Chem. Soc 2018, 140, 11551. 30157372 10.1021/jacs.8b03367

[56] Oller-Salvia B, Chin JW, Angew. Chem. Int., Ed Engl 2019, 58, 10844. 31157495 10.1002/anie.201902658 PMC6771915

[57] Owens AE, Iannuzzelli JA, Gu Y, Fasan R, ACS Cent. Sci 2020, 6, 368. 32232137 10.1021/acscentsci.9b00927 PMC7099587

[58] Van Deventer JA, Yuet KP, Yoo TH, Tirrell DA, ChemBioChem 2014, 15, 1777. 25045032 10.1002/cbic.201402184 PMC4199383

[59] Van Deventer JA, Le DN, Zhao J, Kehoe HP, Kelly RL, Protein Eng. Des. Sel 2016, 29, 485. 27515702 10.1093/protein/gzw029 PMC5081042

[60] Stieglitz JT, Kehoe HP, Lei M, Van Deventer JA, ACS Synth. Biol 2018, 7, 2256. 30139255 10.1021/acssynbio.8b00260 PMC6214617

[61] Stieglitz JT, Potts KA, Van Deventer JA, ACS Synth. Biol 2021, 10, 3094. 34730946 10.1021/acssynbio.1c00370 PMC8936162

[62] Stieglitz JT, Lahiri P, Stout MI, Van Deventer JA, ACS Synth. Biol 2022, 11, 1824. 35417129 10.1021/acssynbio.2c00001 PMC10112046

[63] Stieglitz JT, Van Deventer JA, ACS Synth. Biol 2022, 11, 2284. 35793554 10.1021/acssynbio.1c00626 PMC10065163

[64] Zackin MT, Stieglitz JT, Van Deventer JA, ACS Synth. Biol 2022, 11, 3669. 36346914 10.1021/acssynbio.2c00267 PMC10065164

[65] Islam M, Kehoe HP, Lissoos JB, Huang M, Ghadban CE, Berumen Sánchez G, Lane HZ, Van Deventer JA, ACS Chem. Biol 2021, 16, 344. 33482061 10.1021/acschembio.0c00865 PMC8096149

[66] Alcala-Torano R, Islam M, Cika J, Lam KH, Jin R, Ichtchenko K, Shoemaker C, Van Deventer J, ChemRxiv 2022, 10.26434/chemrxiv-2022-lrj2r. PMC10065152 36459441

[67] Birch-Price Z, Taylor CJ, Ortmayer M, Green AP, Protein Eng. Des. Sel 2023, 36, gzac013. 36370045 10.1093/protein/gzac013 PMC9863031

[68] Drienovská I, Mayer C, Dulson C, Roelfes G, Nat. Chem 2018, 10, 946. 29967395 10.1038/s41557-018-0082-z

[69] Burke AJ, Lovelock SL, Frese A, Crawshaw R, Ortmayer M, Dunstan M, Levy C, Green AP, Nature 2019, 570, 219. 31132786 10.1038/s41586-019-1262-8

[70] Trimble JS, Crawshaw R, Hardy FJ, Levy CW, Brown MJB, Fuerst DE, Heyes DJ, Obexer R, Green AP, Nature 2022, 611, 709. 36130727 10.1038/s41586-022-05335-3

[71] Könning D, Kolmar H, Microb. Cell Fact 2018, 17, 32. 29482656 10.1186/s12934-018-0881-3 PMC6389260

[72] Bowen J, Schneible J, Bacon K, Labar C, Menegatti S, Rao BM, Int. J. Mol. Sci 2021, 22, 1634. 33562883 10.3390/ijms22041634 PMC7915732

[73] Lipovsek D, Lippow SM, Hackel BJ, Gregson MW, Cheng P, Kapila A, Wittrup KD, J. Mol. Biol 2007, 368, 1024. 17382960 10.1016/j.jmb.2007.02.029

[74] Woldring DR, Holec PV, Zhou H, Hackel BJ, PLoS One 2015, 10, e0138956. 26383268 10.1371/journal.pone.0138956 PMC4575168

[75] Kruziki MA, Sarma V, Hackel BJ, ACS Comb. Sci 2018, 20, 423. 29799714 10.1021/acscombsci.8b00010 PMC6051759

[76] Kintzing JR, Filsinger Interrante MV, Cochran JR, Trends Pharmacol. Sci 2016, 37, 993. 27836202 10.1016/j.tips.2016.10.005 PMC6238641

[77] Kimura RH, Levin AM, Cochran FV, Cochran JR, Proteins 2009, 77, 359. 19452550 10.1002/prot.22441 PMC5792193

[78] Silverman AP, Levin AM, Lahti JL, Cochran JR, J. Mol. Biol 2009, 385, 1064. 19038268 10.1016/j.jmb.2008.11.004 PMC2925133

[79] Silverman AP, Kariolis MS, Cochran JR, J. Mol. Recognit 2011, 24, 127. 21194123 10.1002/jmr.1036

[80] Kintzing JR, Cochran JR, Curr. Opin. Chem. Biol 2016, 34, 143. 27642714 10.1016/j.cbpa.2016.08.022

[81] Ravikumar A, Arzumanyan GA, Obadi MKA, Javanpour AA, Liu CC, Cell 2018, 175, 1946. 30415839 10.1016/j.cell.2018.10.021 PMC6343851

[82] Molina RS, Rix G, Mengiste AA, Alvarez B, Seo D, Chen H, Hurtado J, Zhang Q, Donato García-García J, Heins ZJ, Almhjell PJ, Arnold FH, Khalil AS, Hanson AD, Dueber JE, Schaffer DV, Chen F, Kim S, Ángel Fernández L, Shoulders MD, Liu CC, Nat. Rev. Methods Primers 2022, 2, 37. 37073402 10.1038/s43586-022-00130-w PMC10108624

[83] Rix G, Watkins-Dulaney EJ, Almhjell PJ, Boville CE, Arnold FH, Liu CC, Nat. Commun 2020, 11, 5644. 33159067 10.1038/s41467-020-19539-6 PMC7648111

[84] Van Gelder K, Oliveira-Filho ER, García-García JD, Hu Y, Bruner SD, Hanson AD, ACS Synth. Biol 2023, 12, 963. 36920242 10.1021/acssynbio.2c00512 PMC10127261

[85] Wellner A, McMahon C, Gilman MSA, Clements JR, Clark S, Nguyen KM, Ho MH, Hu VJ, Shin J-E, Feldman J, Hauser BM, Caradonna TM, Wingler LM, Schmidt AG, Marks CS, Abraham J, Kruse AC, Liu CC, Nat. Chem. Biol 2021, 17, 1057. 34168368 10.1038/s41589-021-00832-4 PMC8463502

[86] Ravikumar A, Arrieta A, Liu CC, Nat. Chem. Biol 2014, 10, 175. 24487693 10.1038/nchembio.1439

